# An interrater reliability study of gait analysis systems with the dual task paradigm in healthy young and older adults

**DOI:** 10.1186/s11556-021-00271-z

**Published:** 2021-08-03

**Authors:** Thomas Jürgen Klotzbier, Bettina Wollesen, Oliver Vogel, Julian Rudisch, Thomas Cordes, Thomas Jöllenbeck, Lutz Vogt

**Affiliations:** 1 Department of Sport and Exercise Science, University of Stuttgart, Allmandring 28, 70569 Stuttgart, Germany; 2 Department of Human Movement Science, University of Hamburg, Mollerstraße 10, 20148 Hamburg, Germany; 3 Biological Psychology and Neuroergonomics, TU Berlin, Fasanenstr. 1, 10623 Berlin, Germany; 4 Department of Neuromotor Behavior and Exercise, Institute of Sport and Exercise Sciences, University of Münster, Horstmarer Landweg 62B, 48149 Münster, Germany; 5 Institute for Biomechanics, Clinic Lindenplatz, Weslarner Str. 29, 59505, Bad Sassendorf, Germany; 6 Department of Exercise & Health, University of Paderborn, Warburger Straße 100, 33098 Paderborn, Germany; 7 Department of Sports Medicine, Goethe University Frankfurt am Main, Ginnheimer Landstr. 39, 60487 Frankfurt, Germany

**Keywords:** Cognitive-motor interference, Dual task walking, Older adults, Verbal fluency, Minimal detectable change, Gait analysis

## Abstract

**Background and aims:**

One reason for the controversial discussion of whether the dual task (DT) walking paradigm has an added value for diagnosis in clinical conditions might be the use of different gait measurement systems. Therefore, the purpose was 1) to detect DT effects of central gait parameters obtained from five different gait analysis devices in young and old adults, 2) to assess the consistency of the measurement systems, and 3) to determine if the absolut and proportional DT costs (DTC) are greater than the system-measurement error under ST.

**Methods:**

Twelve old (72.2 ± 7.9y) and 14 young adults (28.3 ± 6.2y) walked a 14.7-m distance under ST and DT at a self-selected gait velocity. Interrater reliability, precision of the measurement and sensitivity to change were calculated under ST and DT.

**Results:**

An age effect was observed in almost all gait parameters for the ST condition. For DT only differences for stride length (*p* < .029, ɳ^2^_p_ = .239) as well as single and double limb support (*p* = .036, ɳ^2^_p_ = .227; *p* = .034, ɳ^2^_p_ = .218) remained. The measurement systems showed a lower absolute agreement compared to consistency across all systems.

**Conclusions:**

When reporting DT effects, the real changes in performance and random measurement errors should always be accounted for. These findings have strong implications for interpreting DT effects.

## Introduction

It is well accepted that walking outside of clinical settings requires dual tasking (DT) or multiple-task performance, where walking is combined with cognitive or motor tasks, for example crossing the street while reading signs or observing traffic [1]. Paul, Ada and Canning [2] proposed that there are two reasons why older adults (OA) show decreased performance in multiple-task condition compared to young adults (YA). First, usual physiological changes associated with ageing (decreased muscle mass, visual acuity, changes in proprioception, the vestibular- and somatosensory system) as well as accompanying alterations (postural adjustments, attentional capacity, increased reaction time, etc.) could interfere with DT performance. Second, decrements in physical activity at this age means that multiple-task performance may no longer be a prominent feature of everyday activities [3, 4] and therefore, with the lack of practice, performance declines. Thus, for example, changes in the gait pattern while dual tasking can lead to injourious falls [5] or serious traffic accidents [6], if the attentional resources are not sufficient to process environmental conditions (e.g., a car coming up to cross the road [7];). Dual tasking describes the simultaneous processing of two tasks. In research and clinical settings, the aim of DT paradigms is to calculate proportional dual task costs (DTC) as evidence for the limitation of the information processing system. When calculating DTC, performance in each task under DT condition is related to the respective performance under single task (ST) condition [8]. These proportional DTCs are expressed as a percentage decrease in performance compared to performance in the ST. The term DTC implies that under DT conditions there is an interfering interaction and a deterioration in the processing of the individual tasks, i.e. ST. However, DTs do not always and in all situations lead to performance declines compared to STs, therefore the term “dual task effect” (DTE) or “cognitive-motor interference (CMI)” is more commonly used. We use the term DTC to emphasize the performance decline and DTE to compare the accuracy of different measurement systems.

There are several models that try to explain age related drecrements in DT performance [9, 10]. With the most common resource-theoretical conception of the attention construct [11, 12], it can be assumed that especially in OA with reduced resources for cognitive and motor control, the challenges of dealing with DT are greater [13]. Lindenberger et al. [14] were able to show that the DTC become larger with increasing age. Thus, the cognitive-motor DT gait paradigm can be used to detect gait deficits that would otherwise remain hidden during normal walking without additional tasks. Based on the assumed interrelation of motor- and cognitive function for gait, the DT paradigm is used for diagnosis, prevention and treatment of falls or cognitive impairment (e.g., intervention measures) and there is a controversial discussion of whether such paradigms have an added value [15, 16]. The heterogeneity of the study results can be explained primarily by the choice of cognitive tasks. To address this problem, Al-Yahya and colleagues [17] have published a task classification and were able to show that mental tracking tasks in particular (internal disturbing factors such as counting backwards or verbal fluency tasks) cause significant DTCs on gait. This effect is emphasized in old age and already impaired cognitive abilities.

Regarding walking performance and gait kinematics, most studies currently focus on about eight gait parameters and their variability (gait velocity, cadence, step width, single and double limb support phase, step length, gait cycle length, step duration and gait cycle duration; cf. [18]). These parameters can be classified into parameters of rhythm (e.g., cadence, single and double support) and pace (e.g., gait velocity and step length) [19]. However, different gait analysis systems are used in the various studies to measure these gait parameters, which might limit direct comparability. Only few studies deal with the measurement accuracy of these systems in comparison to established reference systems [20]. Also, regarding the DT gait paradigm, recommendations on methodical procedures regarding walking distance, walking condition (self-selected gait velocity in a slow or fast gait conditions), etc. are rarely provided [16]. Klotzbier and Schott [21] were able to show that especially walking with directional changes is sensitive to the production of DTC. Straight walking does not sufficiently address real-life gait [16]. However, in most studies, walking straight ahead is used as a motor task, as most gait analysis systems are constrained to a straight walkway due to the design of the system (e.g., pressure plates [GAITrite; Zebris] or LED photoelectric switches [OptoGait]). In addition, the algorithms for calculating the gait parameters from the acceleration data of the inertial sensors (GaitUp; MobilityLab) are explicitly and exclusively designed for conditions with straight walking. The different studies use a range of walking conditions, and the measurement range is not always identical [22–24]. With Zebris, for example, it is only possible to cover a range of two meters (OptoGait and GAITrite also have limits). Moreover, one must reflect the algorithms in inertial sensor systems like the MobilityLab that only allow the detection of so-called “steady-state” walking. Also, the algorithms of GaitUp are difficult to comprehend because the raw data cannot be accessed. The question remains unanswered to what extent the common gait parameters of the different gait analysis systems agree despite different measuring principles or walking conditions (ST vs. DT conditions) [24]. Overall, mobile gait analysis systems show excellent agreement for spatiotemporal variables (gait velocity, cadence, gait cycle time, double step time) compared to more elaborate “gold-standard” systems [25–27]. Less agreement has usually been observed for stance-, swing- and double stance phase [25, 27–29]. Although the first direct comparisons of GAITrite and OptoGait [30, 31] as well as GAITrite and MobilityLab [20, 32] resulted in good agreement between systems, no data is available; neither comparisons of other systems, nor for a simultaneous data collection on all systems.

It is crucial that the change of gait parameters from ST to DT is higher than the random measurement error between the systems, especially when retrospectively viewing the effect of DT on gait parameters in meta-analyses or intervention studies. One way to differentiate between real change and random measurement error is through the utilization of the standard error of measurement (SEM) and the minimal detectable change (MDC). Hence, in this study we compared five different gait measurement systems regarding their reliability – in terms of agreement – in a DT paradigm thereby providing an indication of the minimum amount of the DT effect that is necessary to be sure not to consider this as a measurement error.

Therefore, the purpose of the present study was 1) to investigate the average DT effects in a cohort of YA and OA, (i.e., the change in gait parameters from ST to DT), 2) to compare the obtained gait parameters between the measurement systems in YA and OA under ST condition, and 3) to investigate if the DT effects are greater than the measurement error of the systems measured under ST condition. We assumed that DTCs influencing gait parameters would be particularly evident in OA and, that the comparison of the different gait measuring systems indicate no systematic or random differences. Furthermore, we predicted that the DTCs are greater than the average difference of the measurement systems, otherwise the DT effect may be due to measurement error.

## Methods

### Participants

A total of 26 participants were recruited. Community dwelling OA (*n* = 12) who participated in regular fall prevention programs and sports activities for senior citicens at the University of Hamburg and YA (sport students, *n* = 14) were recruited for this study (see Table 1 for group characteristics). All participants had normal or corrected-to-normal vision and no known neurologic or orthopaedic disorder affecting their gait. The study, approved by the local ethics of the University of Hamburg (registration number 2020_2077; 12.2.2020), followed the Declaration of Helsinki [33].

**Table 1:** Sampling characteristics of older adults (OA) and young adults (YA), including mean values (standard deviation) and statistic analyses of the mean value differences

	OA	YA	stat. Analyses
	(*n* = 12)	(*n* = 14)	
**Age** (years)	72.2 ± 7.9	28.3 ± 6.2	*F*(1,24) = 252***, ɳ^2^_p_ = .913
**Sex** (n female)	6	8	CHI^2^(1) = .133^ns^
**Weight** (kg)	74.0 ± .08	72.9 ± 14.2	*F*(1,24) = .045^ns^, ɳ^2^_p_ = .002
**Height** (cm)	1.69 ± .08	1.79 ± .09	*F*(1,24) = 6.16*, ɳ^2^_p_ = .204
**BMI** (kg/m2)	25.1 ± 3.11	22.6 ± 2.75	*F*(1,24) = 6.68*, d = .218
**Leg length left**	83.9 ± 4.79	86.9 ± 6.18	*F*(1,24) = 1.08^ns^, ɳ^2^_p_ = .043
**Leg length right**	−.916 ± 4.67	86.7 ± 6.03	*F*(1,24) = 1.11^ns^, ɳ^2^_p_ = .044
**Shoe size**	41.8 ± 3.08	42.3 ± 3.09	*F*(1,24) = .161^ns^, ɳ^2^_p_ = .007

*ns* Not significant

***p < .001; *p < .05

### Measurement systems

Five different commercially available systems for performing gait analysis in clinical and research settings were compared. We included systems that used external hardware to measure ground contact either through pressure sensors or via optoelectronic devices (body-detached), as well as systems composed of inertial measurement units (body-attached sensors).

In analogy to the study procedure of Rudisch and colleagues [34] we used the same systems. Rudisch et al. compared different outcomes for the overlapping gait phases, during which all systems measured the same steps. The present study focused on the mean values and standard deviation for all walking conditions (see Experimental Setup and Procedure). In the PROCARE multicenter study our group collected data with five different mobile gait measurement systems, according to different institutional resources (see study protocol [35]). The PROCARE project was conducted to develop a training intervention to increase mobility and psychological well-being of nursing home residents. To show effects of the intervention on DT performance (as the DT paradigm is also used in the PROCARE study), it is necessary to secure that training effects exceed the measurement error.

#### Overground walking systems

The *OptoGait* (Microtgate, Bolzano, Italy) system is an optoelectronic measurement system using parallel bars that are positioned on the ground with 0.6 m distance (adjusted to the width of the Zebris plate) and has a spatial and temporal resolution of 1.041 cm (distance between diodes) and 1 kHz respectively. Ground contacts are measured when the photoelectronic bridge between an LED and photodiode is interrupted. We used an OptoGait system of 6 m length. The system showed a high level of correlation with all spatio-temporal parameters (ICCs: 0.79–0.95) [31]. The *GAITrite* (CIR Systems, New Jersey, USA) system is an electronic walkway containing a matrix of pressure sensors. We used an 8.7 m GAITrite walkway with an active measuring range of 7.93 m × 0,75 m and with a spatial and temporal resolution of 1.27 cm (length/width of sensors) and 120 Hz respectively. It is well accepted that the GAITrite mat exhibits excellent reliability for most temporal-spatial gait parameters in both YA (ICCs: 0.83–0.94) and OA (ICCs: 0.82–0.91) [36]. The *Zebris* (zebris Medical GmbH, Isny, Germany) plantar pressure system is, like GAITrite, an electronic walkway containing a matrix of pressure sensors. We used a 2 m × 0.6 m Zebris walkway with a spatial and temporal resolution of 0.85 cm (length/width of sensors) and 100 Hz respectively. Reliability was excellent for gait velocity, cadence, gait cycle time, step and double step length (ICC: 0.93–0.99) and poor for relative stance, swing and double stance phases (ICC: 0.24–0.47) [27].

#### Body-attached inertial sensors

**Fig. 1:**
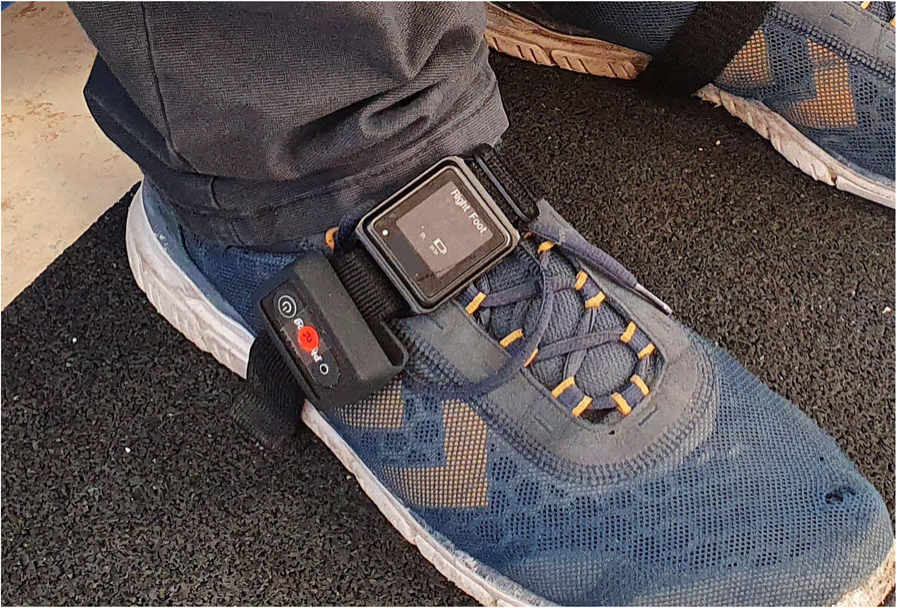
Image showing the attachment of the inertial sensors of GaitUp and MobilityLab

The *GaitUp* is a six-channel inertial sensor system (Physiolog. Lausanne, Switzerland), which is worn on each foot. It is attached to the *MobilityLab* straps and positioned lateral to them on each foot (see Fig. 1). Data was recorded over 14.7 m operating at a sampling frequency of 128 Hz. Moderate to excellent agreement was shown for temporal parameters (ICCs: 0.72–0.97) [29]. The *MobilityLab* (Opal from APDM Inc., Portland, USA) for gait analysis consists of three inertial sensors that are bilaterally attached to both feet with straps and to the fifth lumbar vertebrae. The data recording lasted 14.7 m and sampling at a frequency of 128 Hz. Compared to a treadmill integrated force measuring plate, MobilityLab revealed excellent (ICC: 0.99; range or CI were not reported) agreement for gait velocity, cadence, gait cycle time and double stride length, but only moderate to weak (ICC: 0.50; range or CI were not reported) correlations for stance and swing phase [20].

### Experimental setup and procedure

**Fig. 2:**
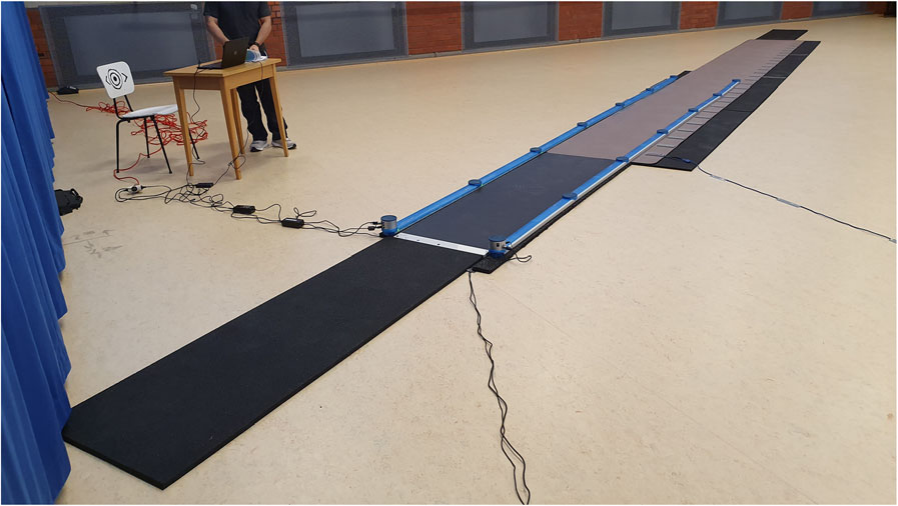
Measurement setup of the overground walking systems Zebris, OptoGait and GAITrite

Figure 2 shows the measurement setup of the systems, illustrating that the gait paths of the different systems overlap only partially. The overall length of the walkway setup was 14.7 m. This is the sum of the distances of the overground walking systems GAITrite (8.7 m) and Zebris (2 m), as well as two mats (each of 2 m length) positioned on both ends of the walkway with the same height as GAITrite, to consider gait initiation and gait termination on an even surface. The walkway (between OptoGait bars) was limited to a width of 0.6 m, corresponding to the width of the Zebris system. Upon arrival in the gym of the Institute of Sports Sciences at the University of Hamburg, where the study was conducted, the participants were informed about the content of the study and signed a declaration of consent. Afterwards the height, weight, leg length (left and right) and shoe size were measured. Then the acceleration sensors were attached (see Fig. 1). Waiting at the starting position the test person was instructed and the walking conditions were described.

### Single task and dual task walking conditions

The subjects had to walk the 14.7 m distance (see Fig. 2) two times without a cognitive task in order to become familiar with the body-attached inertial sensors and another two times (one trial in each condition) for data collection in ST (walking only) and DT condition (walking plus verbal fluency task) in randomized order. In the ST condition the participants were instructed to walk through the walkway at a comfortable, self-selected gait velocity, where in the DT condition the participants should additionally name as many words with a pre-defined letter (B, D, S or A in random order given just before the start signal [35]) as they could think of. The DT condition with an additional verbal fluency task was performed after a short explanation. Participants were allowed to name any word except for proper nouns (such as Bernd or Berlin), numbers, or words that start with the same sound but have a different ending, e.g., love, lover, lovers. These instructions were given and, using a letter that was not assigned by randomization, some examples (3–5 words) were given to ensure that the task was understood. Gait parameters were recorded, and the number of words was counted while walking straight forward. After each trial, the participants were asked to stand still behind the 2 m mat (intended for the deceleration phase) for 5 s so that the accelerometers could transmit their data without interference. Participants were then asked to return to the starting position for the next trial. Data collection for the ST and DT walking conditions was about 10 min.

### Statistical analysis

The mean values and standard deviations of the gait parameters and the respective condition (ST and DT) were considered, using individual data acquisition and analysis software of the respective systems. Six outcome variables were analyzed as they were recorded by every system: velocity (m/s); cadence (steps/min); stride length ([m], distance either foot moves forward); single limb support ([%], time of only 1 foot supporting the body weight); double limb support phase ([%]; ground contact time for both feet); stance phase ([%], duration of ground contact [heel-strike to toe-off]).

Absolute motor DTCs were calculated as follows: (−(STperformance - DTperformance)), negative values indicate decreases from ST to DT. Proportional motor DTCs were calculated as follows: [((DTperformance - STperformence)/STperformance) *100] expressed in % [8, 37]. Since we did not perform cognitive performance under ST condition, it was not possible to calculate cognitive DTCs.

The interrater reliability (ICC) for the comparison within and between the various systems was calculated with the ICC (two-way random model for absolute agreement and for consistency), if an ICC of < 0.5 is bad, 0.5–0.75 is moderate, between 0.75 and 0.90 is good, and greater than 0.90 is excellent [38]. Using the ICC, the standard error of measurement (SEM) and the minimal detectable change (MDC) can be calculated (as preferred statistics according to the COSMIN standards [39]. SEM is an indicator of absolute reliability and precision of the measurement in the same units as the original measurement (SEM = *S*D × √1 – *ICC*). Measurement error was expressed as a percentage of the mean, which was defined as SEM% = (SEM / mean) × 100. A SEM% smaller than 10% indicates excellent agreement or reliability [40]. The MDC (MDC95 = *SEM* × 1.96 × √(2)) can be calculated, where 1.96 derives from the 95% confidence interval of no change and √2 is included because two measurements are involved in measuring change (ST and DT). The MDC is interpreted as the smallest amount of change required to designate a change as real and beyond the bounds of measurement error [41–43], also referred to as the sensitivity to change. Also, the MDC95 was expressed as a percentage, which was defined as MDC95% = (MDC95 / mean) × 100. The mean is the average for all the parameter values in ST [43]. While the ICC ranges from 1 to 0, with 1 being perfect and 0 being no correlation, for good instruments the SEM/SEM% and MDC/MDC95% should be as small as possible.

Power analysis (using G*Power3; a statistical power analysis program [44];) was conducted to estimate the necessary sample size. With a sample size of 16 in one group, an ANOVA with repeated measures would have 80% power to detect the interaction effect size of 0.403 at the 0.05 level of significance. To detect significant group differences (MANOVA between factor: OA vs. YA) of 0.5 or larger (*p* < 0.05) and a power of 80%, twelve participants in each group are necessary. With twelve OA and 14 YA we achieved a total number of 26.

Data were analyzed using SPSS, version 25.0 (SPSS Inc., Chicago, Illinois). To compare the different systems an ANOVA with measurement repetition with the systems as measurement repetition factor was calculated for each gait parameter. To calculate the differences between YA and OA a 6 (gait parameter) × 2 (group) MANOVA was calculated for ST and DT. To calculate the differences between ST and DT, post hoc analyses were calculated for each individual parameter. The mean values of the five systems were used as the basis for the calculation. There were no missing values. If the result of the ANOVAs was significant, post-hoc tests (Bonferoni) were used to analyze which factor levels significantly differed from each other (*p* values set to .05 [45]. Effect sizes for all ANOVAs were reported using the partial Eta^2^ (η^2^_p_).

## Results

### Participants

Table 1 shows the characteristics of the sample. The sex distribution did not differ between the groups. With 1.79 m YA were significantly taller than OA (1.69 m) and had a significantly lower BMI (22.6 ± 2.75) compared to OA (25.1 ± 3.11).

### Age-related differences in gait parameters under single and dual task condition

Overall, an average of 7.39 (SD = 5.75) steps were detected in YA under ST condition and across all systems (GaitUp = 18.1; OptoGait = 5.14; GAITrite = 7.43; Zebris 1.07; MobilityLab = 5.17). Under DT, an average of 10.1 (SD = 4.97) steps was detected (GaitUp = 19.21; OptoGait = 5.64; GAITrite = 8.29; Zebris = 1.29; MobilityLab = 6.08). In OA, an average of 8.89 (SD = 6.10) steps could be detected in the ST (GaitUp = 20.3; OptoGait = 6.33; GAITrite = 8.83; Zebris = 2.25; MobilityLab = 6.71) and an average of 9 (SD = 6.04) steps was detected in the DT condition (GaitUp = 20.1; OptoGait = 6.92; GAITrite = 9.5; Zebris = 2.00; MobilityLab = 6.50).

**Fig. 3:**
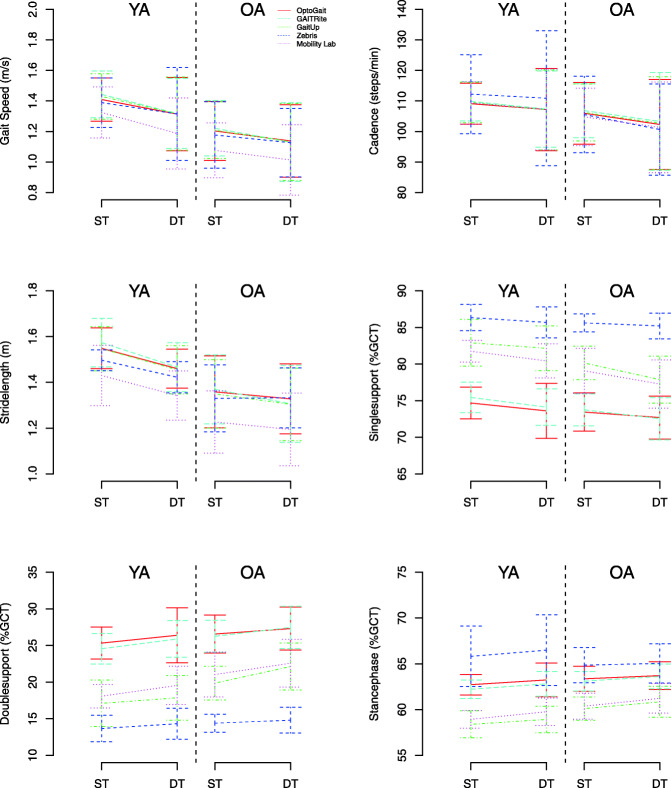
Differences in gait parameters between young adults (YA) and older adults (OA) under ST and DT conditions for all gait measurement systems

The 6 × 2 MANOVA showed that YA and OA differ under ST conditions in gait velocity, *F*(1,20) = 9.11, *p* = .007, ɳ^2^_p_ = .313, stride length, *F*(1,20) = 11.4, *p* = .003, ɳ^2^_p_ = .364, single limb support, *F*(1,20) = 9.99, *p* = .019, ɳ^2^_p_ = .268, double limb support, *F*(1,20) = 10.3, *p* = .004, ɳ^2^_p_ = .340, and in the stance phase, *F*(1,20) = 4.82, *p* = .040, ɳ^2^_p_ = 192. Most gait parameters deteriorated under DT conditions, while differences between YA and OA were found in stride length, *F*(1,20) = 5.66, *p* < .029, ɳ^2^_p_ = .239, single limb support, *F*(1,20) = 4.12, *p* = .036, ɳ^2^_p_ = .22, double limb support, *F*(1,18) = 5.29, *p* = .034, ɳ^2^_p_ = .227, and no significant differences in gait velocity, *F*(1,29) = 3.36, *p* = .082, ɳ^2^_p_ = .144 (see Fig. 3).

Multiple comparisons revealed that differences in gait parameters between ST and DT in YA could only be observed in stride length, *p* = .016. In OA, differences were observed for single limb support, *p* = .014, and double limb support, *p* = .017. In all other gait parameters analyzed, no difference between ST and DT were observed.

### Reliability and minimal detectable changes

The mean values of the different measurement systems under ST condition were compared (cf. Table 2). The relative and absolute reliability measures (ICC^a; c^, SEM, MDC95) are shown in Table 2. The absolut agreement (ICC^a^) between the systems was poor to excellent for all groups and parameters, with values between .255 and .992 [47]. The phase parameters single limb support (.255–.310), double limb support (.272–.309) and stance phase (−.448–.475) in particular showed poor absolute agreement between the systems. The consistency of measurement across all systems (ICC^c^) was moderate to excellent, with values between 0.708 and 0.993. The SEM% was low in all conditions and groups (0.771–4.52%). In 100% of the observations a SEM% ≤ 10% was found. The SEM% varied between 1.09–4.52% for YA and between 0.77–46.4% for OA. The MDC95% was between 2.09–17.8% for all goups and parameters. The MDC95% fluctuated around 17.1% for the total sample. In a variance-analytical comparison of the systems, differences can be reported for almost all parameters.

**Table 2:** Mean values and standard deviation for gait parameters under single task condition (Mean ± SD), and intra-class correlation (ICC), inter-trial reliability (SEM; SEM%) and sensitivity to change (MDC95, MDC95%) for these gait parameters across measurement systems

Parameter	GaitUp Mean (SD)	Opto-Gait Mean (SD)	GAIT-rite Mean (SD)	Zebris Mean (SD)	Mobility Lab Mean (SD)	ICC^**c**^ (95% CI)	ICC^**a**^ (95% CI)	SEM (SEM%)	MDC95 (MDC95%)	ANOVA
**velocity (m/s) YA**	1.47 (0.19)	1.45 (0.19)	1.48 (0.19)	1.44 (0.24)	1.36 (0.19)	0.99 (0.98–0.99)	0.98 (0.93–0.99)	0.02 (1.26)	0.05 (3.49)	F(4,9) = 21.9^***^
**velocity (m/s) OA**	1.23 (0.19)	1.23 (0.20)	1.24 (0.19)	1.19 (0.22)	1.10 (0.19)	0.87 (0.69–0.96)	0.86 (0.68–0.95)	0.08 (6.43)	0.21 (17.8)	F(4,11) = 1.39^ns^
**cadence (steps/s) YA**	111.8 (9.68)	111.5 (9.97)	112.1 (9.35)	117.1 (19.66)	111.6 (9.73)	0.96 (0.91–0.99)	0.96 (0.89–0.98)	2.24 (1.99)	6.22 (5.51)	F(4,9) = 2.29^T^
**cadence (steps/s) OA**	106.9 (9.21)	106.6 (9.86)	107.5 (8.75)	106.3 (12.18)	105.6 (9.42)	0.99 (0.98–0.99)	0.99 (0.98–0.99)	0.82 (0.77)	2.28 (2.14)	F(4,11) = 1.69^ns^
**stride length (m) YA**	1.56 (0.09)	1.56 (0.09)	1.58 (0.10)	1.49 (0.04)	1.44 (0.13)	0.95 (0.88–0.97)	0.88 (0.62–0.97)	0.02 (1.27)	0.05 (3.51)	F(4,9) = 18.1^***^
**stride length (m) OA**	1.37 (0.16)	1.37 (0.16)	1.38 (0.16)	1.35 (0.15)	1.25 (0.14)	0.99 (0.98–0.99)	0.96 (0.84–0.99)	0.02 (1.12)	0.04 (3.11)	F(4,11) = 34.4^***^
**single limb support (%GCT) YA**	82.9 (3.01)	75.1 (2.45)	75.9 (2.59)	86.7 (2.07)	82.1 (1.73)	0.71 (0.27–0.92)	0.26 (−0.01–0.64	0.89 (1.09)	2.45 (3.05)	F(4,9) = 61.9^***^
**single limb support (%GCT) OA**	79.9 (2.33)	73.3 (2.57)	73.5 (2.19)	85.4 (1.32)	79.1 (2.93)	0.81 (0.57–0.94)	0.31 (0.02–0.66)	0.76 (0.98)	2.11 (2.69)	F(4,11) = 105.6^***^
**double limb support (%GCT) YA**	17.0 (3.01)	24.9 (2.45)	24.0 (2.59)	13.3 (2.07)	17.7 (1.88)	0.73 (0.33–0.92)	0.27 (−0.01–0.66)	0.88 (4.52)	2.43 (12.5)	F(4,9) = 63.4^***^
**double limb support (%GCT) OA**	20.1 (2.33)	26.7 (2.57)	26.5 (2.19)	14.6 (1.32)	20.9 (2.93)	0.81 (0.56–0.94)	0.31 (0.02–0.66)	0.76 (3.51)	2.12 (9.72)	F(4,11) = 104.9^***^
**stance (%GCT) YA**	58.4 (1.39)	62.5 (1.27)	61.9 (1.29)	66.7 (4.19)	58.8 (1.06)	−7.49 (−20.2–1.41)^§^	−0.45 (−0.54–0.16)^§^	1.08 (1.75)	2.99 (4.85)	F(4,9) = 19.5^***^
**stance (%GCT) OA**	60.2 (1.23)	63.5 (1.36)	63.2 (1.09)	65.1 (2.08)	60.4 (1.35)	0.82 (0.59–0.94)	0.46 (0.07–0.79)	0.47 (0.76)	1.31 (2.09)	F(4,11) = 49.3^***^

*YA* Young adults, OA Older adults, *GTC* Gait cycle time, *ICC* Intra-class-correlation, A absolute agreement, C consistency, *95% CI* 95% confidence interval; §The Tukey Additivity Test shows a significant interaction effect between the systems and the persons being assessed, which contradicts the requirements for the ICC analysis. An interpretation is not possible due to a possible under- or overestimation and since reliability measures are by definition limited to a value range from 0 to 1, negative ICCs indicate a reliability of 0 [46]; *SEM* Standard error of measurement, mdc minimal detectable change. In order to be able to compare both measures, they were additionally expressed as percentages (SEM% and MDC95%). ANOVA to calculate the differences between the measurement systems: *T* Tendency, ns Not significant, *p < .05, **p < .01, ***p < .001

### Comparison between real modification and random measurement error

Table 3 shows the motor DTC of the six gait parameters for the two groups separately as well as the smallest amount of change required to designate a change as real and beyond the bounds of measurement error. It can be observed that the percentage DTC was lower than the MDC in percentage for most of the gait parameters in both YA and OA. Especially the low sensitivity of change detection for gait velocity in OA with MDC95% of 17.8% is noticeable.

**Table 3:** The comparison between real modification in performance and the contribution of random measurement error

Parameter	ST Mean (SD)	DT Mean (SD)	DT effect ∆ (DTE%)	DT increase/decline* N (%)	SEM (SEM%)	MDC95 (MDC95%)	Real modification
**velocity (m/s) YA**	1.44 (.198)	1.31 (.237)	−0.13 (− 9.03)	22.2	0.02 (1.26)	0.05 (3.49)	yes
**velocity (m/s) OA**	1.18 (.207)	1.11 (.237)	−0.07 (− 5.93)	36.4	0.08 (6.43)	0.21 (17.8)	no
**cadence (steps/s) YA**	112.8 (11.5)	109.9 (14.2)	−2.9 (− 2.57)	22.2	2.24 (1.99)	6.22 (5.51)	no
**cadence (steps/s) OA**	106.5 (9.81)	102.1 (14.9)	−4.4 (−4.13)	36.4	0.82 (0.77)	2.28 (2.14)	yes
**stride length (m) YA**	1.53 (.088)	1.44 (.094)	−0.09 (−5.88)	66.7	0.02 (1.27)	0.05 (3.51)	yes
**stride length (m) OA**	1.34 (.151)	1.29 (.151)	−0.05 (−3.73)	75.0	0.02 (1.12)	0.04 (3.11)	yes
**Single limb support (%GCT) YA**	80.6 (1.63)	78.9 (2.11)	−1.7 (−2.11)	11.1	0.89 (1.09)	2.45 (3.05)	no
**single limb support (%GCT) OA**	78.3 (1.77)	77.1 (2.17)	−1.2 (− 1.53)	27.3	0.76 (0.98)	2.11 (2.69)	no
**double limb support (%GCT) YA**	19.4 (1.69)	21.0 (2.13)	1.6 (8.25)	88.9	0.88 (4.52)	2.43 (12.5)	no
**double limb support (%GCT) OA**	21.8 (1.76)	22.9 (2.18)	1.1 (5.05)	81.8	0.76 (3.51)	2.12 (9.72)	no
**stance (%GCT) YA**	61.7 (.369)	61.1 (4.23)	−0.6 (−.972)	22.2	1.08 (1.75)	2.99 (4.85)	no
**stance (%GCT) OA**	62.5 (1.11)	61.7 (4.16)	−0.8 (−1.28)	45.5	0.47 (0.76)	1.31 (2.09)	no

The mean values of the measurement systems for the gait parameters divided into YA and OA in ST and DT condition are shown. *YA* Young adults, *OA* Older adults, *GTC* Gait cycle time, *ST* single task, *DT* dual task. ∆ = difference between ST and DT [were calculated as follows: (−(STmean – DTmean)). negative values indicate decreases from ST to DT]. DTC% = proportional dual task costs [were calculated as follows: ((DT - ST) / ST) *100]. *DT increase/decline = % of individuals showing higher values under DT compared to ST condition (whether this is an improvement or a deterioration in performance depends on the gait parameter; for example, for velocity we observe that the value increases for 22.2% of the YA in the DT. Accordingly. the velocity decreases for 77.8%, what is to be expected for the gait velocity. SEM = standard error of measurement of the gait systems. *MDC* Minimal detectable change. Real modification = MDC95% < DTC%

## Discussion

The aim of the study was to detect the amount for DT decrements for OA and YA across five gait analysis systems and to determine wheter the DT effect is greater than the measurement error between these systems. This is important in order to interpret the DTC for example in age comparisons or future training studies.

The main findings for the age comparison of this study were that OA and YA differ under ST conditions in gait velocity, stride length, single and double limb support as well as the stance phase. OA walk slower with shorter stride lengths and lower times in the single limb support phase, but with higher times in the double limb support phase and stance phase. Regarding changes from ST to DT, YA only demonstrated reductions in stride length and OA demonstrated longer single and double limb support times.

Overall, our results show the expected differences in walking performance between OA and YA as ageing is associated with many changes in the locomotor system [48, 49]. It is well described that neural reflexes, visual and vestibular feedback decrease with age [50] and in combination or interaction, these age-related changes lead to decrements of the locomotor coordination and have an impact on walking performance because of decreasing gait stability [51]. We found consistent results with previous studies for reduced gait velocity [52], reduced step length [49] as well as increased double limb support [53] for OA in comparison to YA. Also, most of the studies focusing on falls prevention reported higher decrements of gait parameters for fallers in comparison to non fallers including gait velocity, step length, step width and double limb support time over several years [53] or under DT conditions [54, 55]. Consistent with the literature and according to our results only single and double limb support phase show significant changes. Interestingly, following the classification by Beauchet and colleagues [19], YA only showed DT decrements for parameters of pace whereas OA showed DTC for relevant parameters of pace and rhythm (e.g., velocity and step length; rhythm: double support time). The DTC for both aspects of gait quality might be one explanation for greater gait instabilities of OA.

On the other hand, some studies showed that there is not always a deterioration in performance under DT conditions [54, 56]. According to the “Constrained-Action” hypothesis [57], focusing attention on a highly automated movement (internal focus) leads to performance limitations. In contrast, an external focus of attention towards the cognitive task leads to a self-organized and automated motion sequence and improved performance. As cognitive demands increase, the negative effect of competition for limited attention resources and the beneficial effect of an external attention focus overlap. A comparison between different age groups shows a decreasing positive effect of an additional cognitive task in older persons [58, 59]. Since we neither manipulated the difficulty level of the cognitive task nor calculate proportional cognitive DTC, we cannot confirm the predictions of the “Constrained-Action” hypothesis [57].

The second aim of our study was to analyze the effect of different measurement conditions. Within this study, the variables of rhythm that described the main significant differences between OA and YA for ST conditions (single and double limb support as well as stance phase) showed the poorest absolute agreements between the systems. Therefore, a direct comparison of these parameters of different studies with different systems is only possible to a limited extent. The SEM% was low (0.76–6.43%) in all conditions and both age groups. In 100% of the observations a SEM% ≤ 10% was found. Overall, the MDC95% values ranged from 2.09 to 17.8%. In line with previous results the SEM values of basic spatiotemporal parameters (step length and gait velocity) were lower than values of relative phase parameters (i.e., double support time) [30, 34, 60]. The accuracy of the measurement of spatiotemporal gait parameters of rhythm depends on the precision of the heel strike and toe off detection [61]. A greater variance in the section of these two parameters might also lead to a greater variance of the calculations with the implemented algorhythms. This might be an explanation for the higher SEM values in the gait parameters of rhythm.

Thirdly, we wanted to investigate the minimum required magnitude of change between ST and DT performance to ensure that the gait systems detect a real modification and to be 95% certain that it is not a measurement error. The main results showed that for the relative phase parameters single limb support, double limb support and stance, the DTE is lower than the minimum required magnitude of change according to the MDC95 (calculated based on the SEM). The relatively low agreement in the values for the phase parameters was already observed by Rudisch and colleagues [34], who were able to show that the basic spatiotemporal parameters (i.e., stride length, cadence, and gait velocity) showed better agreement than measures of relative phase parameters (i.e., single support phase, double support phase, stance; see also [20, 27, 30, 31]).

Thus, when interpreting a change in DT studies with walking and under consideration of the different measuring systems, a change in velocity of 0.21 m/s in OA may be considered a real change and indicates that change is not the result of measurement error. It is possible to state with 95% certainty that the change is reliable rather than measurement error, if the absolute and propotional motor DTC for gait velocity are at least 17.8%. These findings have particularly strong implications for the interpretation of study results or to describe training effects on DT performance. Therefore, in line with recommendations by Wollesen and colleagues [16] meta-analysis that deals with gait parameters, the results must consider that the different systems measure with different accuracy.

### Limitations

A limitation in the context of this study is that the setup and positioning of the five measuring systems prevent the overlapping areas being maintained over the entire walking distance. This is especially the case with the overground walking systems, as they have different measuring ranges. We consider the Zebris with a length of two meter as comparatively short, which limits its suitability for overground gait analysis (on a treadmill, the two-meter system can be quite useful). OptoGait and GAITrite are limited by constraints including the length of the walkway, and the suitability for only flat surfaces, however these devices deliver very accurate results. MobilityLab and GaitUp are ecologically valid as they do not constrain the gait of participants and the entire walking distance could be recorded, even though a participant has to get used to the sensors. They do provide good accuracy for basic spatiotemporal parameters but are limited with respect to parameters of relative phase. When comparing MobilityLab and GaitUp, the former filters all steps that are not representative of a “steady-state” walking, which makes accurate step detection impossible. Moreover, GaitUp does not allow access to the raw data, so the data cannot be extracted or analyzed independently.

The study meets the standards for excellent quality according to COSMIN (COnsensus-based Standards for the selection of health Measurement Instruments [39];). The general requirements for studies that use item response theory (IRT) models, the general design issues, and questions regarding reliability (with its measurement properties: reliability and measurement error) are fulfilled. However, the COSMIN recommendations that advise a sample size of 50 ([39], see also [62]) were not met in this study. Thus, the results on the consistency of the measurement systems and the results regarding the comparison between DTE and system-measurement error under ST condition must be interpreted with caution. No firm conclusion can be made for these aims of the study, due to the small sample size.

In addition to the two trials under ST condition to get used to the attached sensors, further DT familiarization trials would have been useful to get used to this condition (specifically related to the additional verbal fluency task). Multiple trails in both conditions could quite possibly increase the validity of the study. Since the performance of the cognitive task under ST condition was not recorded, a conclusion about the cognitive DTC is not possible. It can be assumed that differences in the DTCs are more pronounced in persons with impairments [62]. In this respect, it is uncertain whether a reliable detection of the steps is possible for persons with severe locomotion problems. Especially with overground walking systems it can be difficult to detect a gait characteristic with small shuffling as we see for example in people with Parkinson’s disease or fragile OA.

## Conclusions

It seems important that studies on gait parameters in motor-cognitive DTs provide information not only on the significance and statistical DTC, but also on the probable cause of all reported changes in performance, i.e., on the contribution of both real changes in performance and random measurement errors to the reported changes.

For studies which are to be compared directly with each other and in those where different systems are used, the comparability of the gait parameters must be queried due to the low absolute agreement (absolute reliability). If, on the other hand, intervention effects on gait parameters from different studies are compared with each other, the “acceptable” consistency across the measurement systems ensures comparability and is less problematic. When it comes to the choice of an appropriate measurement system, this must always be seen in relation to the research question, the walking task to be performed and the respective setting in which the systems are used, clinical routine or scientific interest.

## Data Availability

Data can be obtained from the corresponding author upon reasonable request.
