# Correction: An interrater reliability study of gait analysis systems with the dual task paradigm in healthy young and older adults

**DOI:** 10.1186/s11556-022-00301-4

**Published:** 2022-10-05

**Authors:** Thomas Jürgen Klotzbier, Bettina Wollesen, Oliver Vogel, Julian Rudisch, Thomas Cordes, Thomas Jöllenbeck, Lutz Vogt

**Affiliations:** 1 Department of Sport and Exercise Science, University of Stuttgart, Allmandring 28, 70569 Stuttgart, Germany; 2 Department of Human Movement Science, University of Hamburg, Mollerstraße 10, 20148 Hamburg, Germany; 3 Biological Psychology and Neuroergonomics, TU Berlin, Fasanenstr. 1, 10623 Berlin, Germany; 4 Department of Neuromotor Behavior and Exercise, Institute of Sport and Exercise Sciences, University of Münster, Horstmarer Landweg 62B, 48149 Münster, Germany; 5 Institute for Biomechanics, Clinic Lindenplatz, Weslarner Str. 29, 59505 Bad Sassendorf, Germany; 6 Department of Exercise & Health, University of Paderborn, Warburger Straße 100, 33098 Paderborn, Germany; 7 Department of Sports Medicine, Goethe University Frankfurt am Main, Ginnheimer Landstr. 39, 60487 Frankfurt, Germany

**Correction: Eur Rev Aging Phys Act 18, 17 (2021)**

**https://doi.org/10.1186/s11556-021-00271-z**

In the publication of the original article [1] the authors state that the GaitUp system does not allow access to the raw data: “[…] the algorithms of GaitUp are difficult to comprehend because the raw data cannot be accessed” (page 2) and “[…] GaitUp does not allow access to the raw data, so the data cannot be extracted or analyzed independently (page 9).

Since the Physilog 5 launch in 2017, GaitUp has provided the free “Research Toolkit” to its users. This has always been a free on-demand service for all customers (who are only equipped with Physilog 5 or related products such as the gait analysis system used in our study - which was acquired from the University of Paderborn in 2018). Therefore, our statement that the raw data of the acceleration values cannot be accessed, extracted, or analyzed is not correct.

For this reason, the false statements have been deleted and the original article [1] has been updated.
